# Graves Disease Induced by Radioiodine Therapy for Toxic Nodular Goiter: A Case Report

**DOI:** 10.4274/mirt.74046

**Published:** 2015-11-02

**Authors:** Yakup Yürekli, Arzu Cengiz, Engin Güney

**Affiliations:** 1 Adnan Menderes University Faculty of Medicine, Department of Nuclear Medicine, Aydın, Turkey; 2 Adnan Menderes University Faculty of Medicine, Department of Endocrinology, Aydın, Turkey

**Keywords:** Nodular goiter, Graves’ disease, Iodine radioisotopes

## Abstract

Graves’ disease (GD) may be observed as an infrequent adverse effect after radioiodine therapy (RAIT) for toxic thyroid adenoma (TA) and toxic multi nodular goiter (MNG). We present a case of a 55-year-old male with a toxic nodule who was treated with RAI. After therapy, the patient’s serum free triiodothyronine (fT3) and free thyroxine (fT4) levels gradually increased. Antithyroid peroxidase (TPOAb), antithyroglobulin (TgAb) and TSH-receptor antibodies (TRAb) were also positive. Thyroid scintigraphy revealed diffuse intense uptake after four months of RAIT. Radiation-induced GD should be considered in patients with aggravated hyperthyroidism 3-4 months after therapy.

## Abstract (tr)

Toksik adenom (TA) ve multi nodüler guatr (MNG) tedavisi için radyoaktif iyot (RAİ) uygulanmasını takiben nadir bir yan etki olarak Graves hastalığı görülebilir. Bu olguda RAİ ile tedavi edilen, toksik nodülü olan 55 yaşındaki bir erkek hasta sunulmaktadır. Tedavi sonrası hastanın serbest triiyodotironin (fT3) ve serbest tiroksin (fT4) düzeylerinde giderek artış görüldü. Antitiroid peroksidaz (TPOAb), antitiroglobulin (TgAb) ve TSH-reseptor antikorları (TRAb) pozitif idi. RAİ tedavisinden 4 ay sonra yapılan tiroid sintigrafisi diffüz artmış aktivite tutulumu gösterdi. Tedaviden 3-4 ay sonra hipertiroidi bulgu ve belirtilerinde belirginleşme olan hastalarda radyasyona bağlı Graves hastalığı göz önünde bulundurulmalıdır.

## INTRODUCTION

Radioiodine therapy (RAIT) is being increasingly used in patients with toxic thyroid adenomas (TA) and multi nodular goiter (MNG). Radiation thyroiditis is a well-known complication that presents with neck pain, dysphagia, thyroid tenderness and transient hyperthyroidism. This adverse effect usually occurs a few days after RAIT (1,2). Graves’ disease (GD) is reported as an uncommon adverse effect after RAIT for TA and MNG, and usually manifests 3 to 6 months after therapy (3,4,5). The incidence of GD following RAIT for MNG was reported as 5%, and this rate is increased upto 22% in patients who are positive for anti-thyroid peroxidase antibody (TPOAbs) before treatment (2,3).

Herein, we present a patient with TA who developed GD after treatment with RAI.

## CASE REPORT

**Figure 1:**
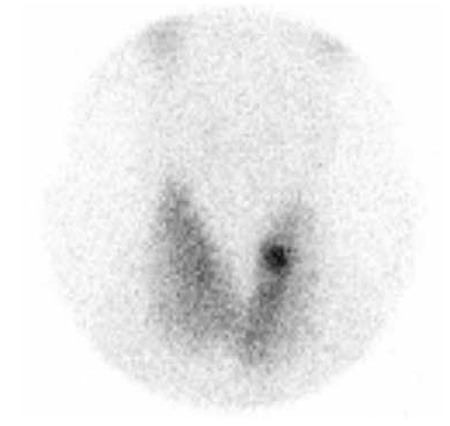
Pre-treatment thyroid scintigraphy showing hyperactive nodule on the left lobe and mild suppression in the remainder of the gland

**Figure 2:**
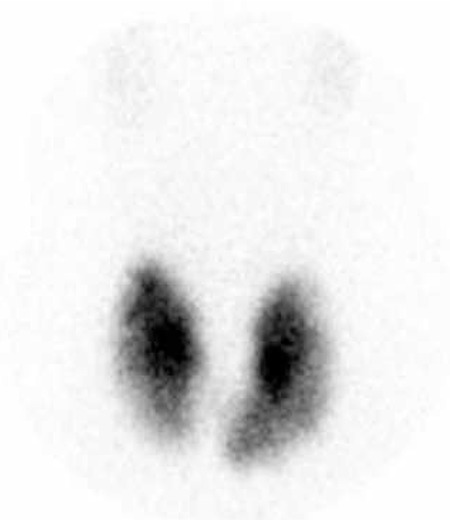
Thyroid scintigraphy 4 months after radioiodine therapy showing diffuse increased uptake

A 55-year-old male patient with subclinical hyperthyroidism and TA was referred to our department for RAIT. Baseline fT3 was 4.01 pg/ml (N: 1.82-4.62 pg/ml), fT4 was 1.61 ng/dL (N: 0.932-1.71 ng/dL) and TSH was 0.167 µIU/ml (N: 0.270-4.2 µIU/ml). Thyroid autoantibodies were not evaluated before RAIT. Physical examination revealed diffuse goiter. Ultrasonography showed an enlarged gland and a 13 mm isoechoic nodule in the left lobe with a few millimetric nodules in the right lobe. Fine-needle aspiration biopsy was interpreted as benign. Thyroid scintigraphy revealed an enlarged gland and a hyperactive area corresponding to the left lobe nodule and mild suppression in the remainder of the gland (Figure 1). RAIT was planned as soon as possible due to his cardiac problems. He was treated with a fixed dose of 10 mCi of I-131. His thyroid function tests in the post-treatment follow-up are shown in Table 1. TPOAbs, TgAbs and TRAbs were positive 4 months after therapy. The patient complained of irritability and anxiety. Thyroid scintigraphy showed an enlarged gland with diffusely increased uptake (Figure 2). Although spontaneous development of GD cannot be excluded, we believe that the autoimmune response of thyroid follicular cells after RAI treatment resulted in GD.

## LITERATURE REVIEW AND DISCUSSION

The transition of a toxic nodular goiter into an autoimmune toxic goiter as a side effect of RAIT has previously been described in case reports, as well as prospective and retrospective studies (2,3,4,6,7). This side effect occurs in as many as 5% of patients with nodular goiter who were treated with I-131 (2). Several pathophysiological mechanisms were suggested to explain the development of autoimmune hyperthyroidism after RAIT. It has been reported that RAIT induced an increase in thyroid-stimulating immunoglobulins within the circulation (1,8). Another mechanism is radiation-induced impairement of immune balance between T-helper and suppressor lymphocytes (9).

Schmidt et al. (10) concluded that patients with elevated TPOAbs before RAIT had an almost 10-fold higher risk of developing post-therapy immunogenic hyperthyroidism. In another study, authors indicated that patients with high serum TPOAb levels before RAIT seem to be at increased risk of developing delayed TRAb-associated hyperthyroidism as well as hypothyroidism. Therefore, they suggested that I-131 treatment should be used in selected patients with elevated TPOAb levels (1).

Although such cases are rare, the occurrence of GD by destruction of follicular cells have also been described after percutaneous ethanol injection, external radiation for nonthyroidal diseases, subacute thyroiditis, and surgical resection of autonomous adenoma or parathyroidectomy (11,12,13,14,15).

It is concluded that physicians should recognize GD as a side effect of RAIT for toxic nodular goiter. The risk assessment should include evaluation of pre-treatment TPOAb levels. Patients with increasingly evident hyperthyroidism 3-4 months after RAIT should be investigated for the development of GD.

## Figures and Tables

**Table 1:**
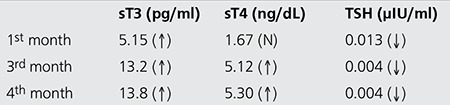
Results of thyroid function tests in the post-treatment follow-up
